# A parasitic patch loaded staircase shaped UWB MIMO antenna having notch band for WBAN applications

**DOI:** 10.1016/j.heliyon.2023.e23711

**Published:** 2023-12-17

**Authors:** Tanvir Islam, Esraa Mousa Ali, Wahaj Abbas Awan, Mohammed S. Alzaidi, Thamer A.H. Alghamdi, Moath Alathbah

**Affiliations:** a Department of Electrical and Computer Engineering, University of Houston, Houston, TX, 77204, USA; b Faculty of Aviation Sciences, Amman Arab University, Amman 11953, Jordan; c Department of Information and Communication Engineering, Chungbuk National University, Cheongju 28644, South Korea; d Department of Electrical Engineering, College of Engineering, Taif University, P.O. Box 11099, Taif 21944, Saudi Arabia; e Wolfson Centre for Magnetics, School of Engineering, Cardiff University, Cardiff CF24 3AA, UK; f Department of Electrical Engineering, College of Engineering, King Saud University, Riyadh 11451, Saudi Arabia

**Keywords:** Body area network, MIMO, Notch band, Staircase antenna, UWB

## Abstract

A staircase-shaped quasi-fractal antenna is presented to meet the requirements of compact electronics operating in UWB or E-UWB spectrum. A conventional broadband monopole antenna is converted into UWB antenna utilizing three iterations of fractal patches. The resultant antenna offers wide impedance bandwidth ranges 2.3–17.8 GHz, having a notch band at 6.1–7.2 GHz. Afterwards, a two-port MIMO antenna is created by placing the second element orthogonally with an edge-to-edge distance of 8.5 mm, that is λ/15 where λ corresponds to free space wavelength at the lowest cut-off frequency. Hereafter, a meandered line-shaped stub is inserted to reduce the mutual coupling between closely spaced MIMO elements to less than −25 dB. As the intended application of the proposed work is On-body, Specific Absorption Rate (SAR) analyses are carried out at 2.4, 5.8 and 8 GHz, showing an acceptable range for both 1-g and 10-g averaged tissues standards. Moreover, various parameters of the MIMO antenna are studied, and a comparison is made between simulated and measured results as well as those of the state of the art.

## 1 introduction

Antennas are required to have a compact size, low–cost and high performance in modern 5G and future 6G communication systems [1]. The performance in terms of high data rate, low latency rate and long battery life is required to facilitate multiple users simultaneously. These changes and market demands have revised the communication model design [2,3]. As the antenna is one of the key parameters of the communication system, the requirements to design antennas are also revised due to revisions in the communication system. The compact antenna, with a low–profile and simplified geometry, is required with wideband and high gain and efficiency [4]. Multi–input multi–output (MIMO) antennas are a good candidate for 5G and 6G applications [5]. MIMO antennas have the advantages of high data rate and low latency. The isolation or mutual coupling between antenna elements is a crucial parameter to examine in MIMO antennas [6,7]. The required value of isolation is < –20 dB, which is challenging to achieve, due to closely spaced antenna elements [8].

To improve isolation, the researcher has introduced a number of approaches, including using parasitic patches between antenna elements, using Defective Ground Structures (DGS), loading Electromagnetic Band Gaps (EBGs) and loading meta–surfaces as well as filters [[9], [10], [11]]. It is important to note that the applications of EBGs and meta-surfaces are not limited to isolation alone, but also include gain enhancement [12,13], phase correction [14], leaky-wave antennas [15], spatial filters [16,17], transmit arrays [18,19] and harmonic suppression [20]. In literature, there are many designs suggested to operate in lower frequency bands ranging from 1.5 to 18 GHz, which includes GPS, ISM, WLAN, WiMAX, 5G–Sub6GHz, C–Band, X–Band and S–Band [[21], [22], [23], [24], [25], [26], [27], [28], [29], [30]]. Some of these designs have complex geometry with the placement of the patches on different layers of the substrate [22], while others with simplified geometry have a narrow band and low gain [25,26]. The researcher also used meta-surfaces to get wide band and high gain to improve the antenna performance, but the overall size is enhanced, and complexity is introduced [27]. Moreover, the other antennas have limitations of narrow bandwidth, low gain and also limited to operate only on some particular applications of ISM and WLAN [28,29].

To overcome these challenges, a wideband antenna is introduced, which has a large size and complex geometry [30]. In order to fulfil the requirements of present and future communication systems, numerous MIMO antennas with improved isolation are proposed in the literature [[31], [32], [33], [34], [35], [36], [37], [38], [39], [40], [41], [42]]. In Ref. [31], a wideband CPW feed patch antenna operating over 3–12 GHz is reported for wireless applications. Although the reported antenna is functional over a wide frequency range and offers a high peak gain of 6.64 dBi, the size is large. This antenna offers −21 dB isolation, which is obtained by introducing a parasitic decoupler. A multi-stub-loaded antenna with DGS ground for improved isolation is provided in Ref. [32]. The antenna has a compact size of 38 mm × 38 mm × 1.6 mm but offers a low isolation value of < −18 dB.

In the literature, numerous MIMO antennas are proposed, which offer UWB along with a notch band [[33], [34], [35], [36], [37], [38], [39], [40], [41], [42], [43]]. A UWB antenna operating on 3–14 GHz is presented in Ref. [33]. The UWB and notch bands are achieved after etching multiple U-shaped slots into a rectangular patch. The antenna has a large size of 82 mm × 40 mm and an unstable radiation pattern due to slot etching. A compact and UWB MIMO antenna given in Ref. [34] offers 2.8–11 GHz. The antenna contains CSRR and DGS ground planes to get UWB, notch band, and isolation enhancement. The setback of this work is the complex geometry and value of ECC (0.02). A simple geometry and compact-sized antenna are presented in Ref. [35]. The antenna contains a simple circular patch and a DGS ground plane. The limitation of this work is the large value of ECC 0.01 and the fact that it didn't provide information about important MIMO parameters. A quad-element UWB MIMO antenna having a notch band is reported in Ref. [36]. The antenna has overall measurements of 60 mm × 60 mm × 1.6 mm and operates on 4–18 GHz with a peak gain of 6.2 dBi. The value of isolation between MIMO elements is 17.5 dB, and another work with a triple notch band has an isolation of 15 dB [37]. These values of isolation are not in the desired range. The value of isolation or mutual coupling must be below 20 dB for MIMO antennas [38,39].

In [40], a compact antenna with a size of 21.5 mm × 28 mm is given for UWB applications. Although the antenna is compact in size and operates at UWB with a notch band, it has an unsatisfactory value of mutual coupling of 16 dB. The isolation or mutual coupling between MIMO antenna elements is improved in Ref. [41] by introducing mushroom EBG. The antenna offers UWB of 3–10.7 GHz, with isolation and gain of 25 dB and 5 dB, respectively. Another high-gain and wide-band antenna is given in Ref. [42]. The antenna has a size of 60 mm × 60 mm, a bandwidth of 3.9–12 GHz, and a gain of 6.94 dB. Although the antenna has high gain, wideband, and simple geometry, it is large. In Ref. [43], a UWB antenna offering 2.4–13 GHz is given. The antenna has a size of 50 mm × 82 mm and a peak gain of 6 dB. The antenna offers high gain and a wide band but has a large size and complex geometry.

**Fig. 1:**
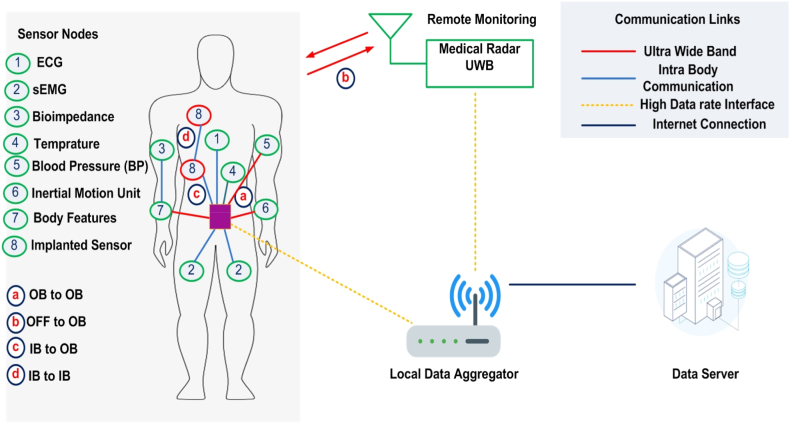
Connection of the human body with various wireless sensors in body area network applications.

With the development of technology, the connection of humans with wireless devices is increasing daily. In the near future, it is predicted that one person will be connected to several wireless sensors for various applications [44]. It can be seen from the model given in Fig. 1, a person can be connected for health care, sports, and daily activity purposes. The figure also clearly shows the connection between body sensors and the internet, along with the role of the antenna. It is clear that antenna operating over UWB also has a key role in ON body applications, as it helps greatly to optimize a transceiver for MIMO communication systems [45,46]. In the literature, the design for the antenna is presented and measured the results for ON body applications as well.

A broadband antenna operating over 1.4–5 GHz is presented in Ref. [47]. The antenna has complex and large measurements of 140 mm × 80 mm, with peak gain of 2.9 dBi. A compact and simplified geometrical antenna is given in Ref. [48]. The antenna has 36 mm × 48 mm dimensions, with a peak gain of 7 dBi. The AMC structure is loaded to im-prove the gain, but this approach introduces complexity. In Ref. [49], an antenna, a UWB antenna operating over 3.7–10.3 GHz is presented. The design is engineered over PDMS material for ON body applications. The antenna has flexibility and UWB but complex geometry as well as a large size of 80 mm × 67 mm. A UWB MIMO antenna in Ref. [50] operates over 2–14 GHz and offers gain of 7.2 dBi. The antenna has UWB and high gain, but low isolation of – 15 dB. Finally, a simplified geometrical antenna in Ref. [51], which has a compact size of 30 mm × 18 mm is presented for ON body applications. The antenna has the demerit of narrow bandwidth.

From the above literature assessment, it is obvious that more research is needed to create antennas with small sizes, easy geometries, and low profiles that provide wideband, high gain, and low isolation. This research suggests an antenna to solve the aforementioned characteristics. The recommended antenna has novelty of.

1) Compact size and simple structure.
2) Operating over UWB spectrum along with Notch band.
3) Offers high value of gain.
4) Suitable for wireless sensor networks
5) MIMO configuration with acceptable range of parameters.
6) Novel antenna shape along with novel structure of parasitic patch used to improve the isolation of antenna.

Four sections make up the remaining article. The description of a single element, design phases, outcomes, and parametric analysis, follows. The MIMO antenna is explained with performance characteristics in the third part. The last section concludes the proposed work with a comparison table and references.

## 2 Design of notched UWB antenna

The proposed ultra-wideband antenna design, along with its design procedure, is discussed in this section. The results for the single element in the form of scattering parameter, gain vs frequency plot and radiation pattern are discussed. Afterwards the MIMO design approach with improved isolation is also discussed in the next section.

### 2.1 Geometrical configuration

**Fig. 2:**
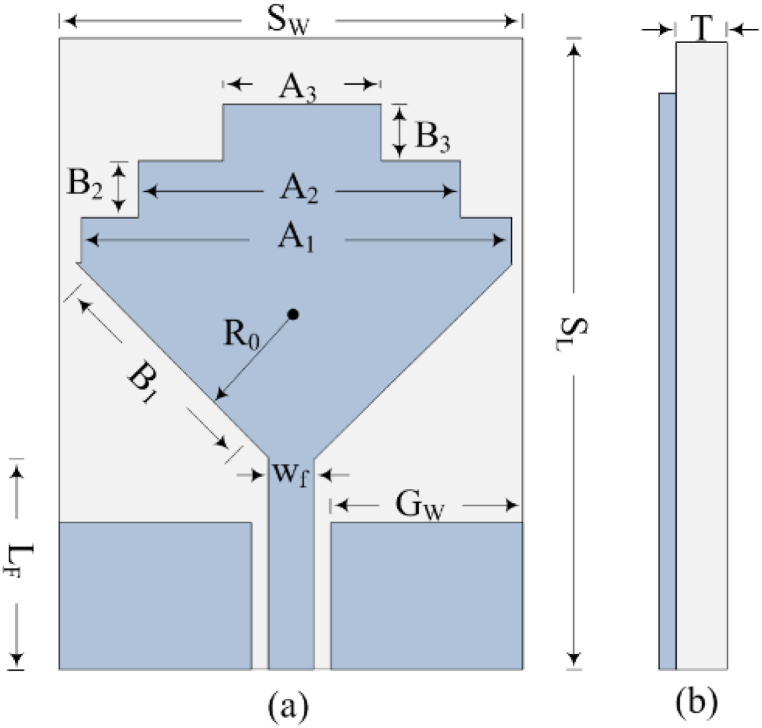
The geometrical configuration of dual staircase antenna (a) top-view (b) side-view.

The structural arrangement with labelled optimized parameters is illustrated in Fig. 2(a and b). The suggested design contains a CPW feed line loaded with a dual staircase-shaped radiator. Three consecutive iterations are performed by loading rectangular stubs to upgrade return loss and enhance the bandwidth. The CPW feedlines are deployed with the supremacy of low dispersion and ease of fabrication. The antenna has an overall size of S_L_ × S_W_ × T = 34 mm × 25 mm × 1.52 mm. The radiated element is loaded on the front side of commercially available substrate material Rogers RT/Duroid 6002 having relative permittivity and loss tangent of 2.94 and 0.0012, respectively.

The optimized dimensions of various dual staircase shaped antenna are as follows S_L_ = 34; S_W_ = 25; A_1_ = 23; A_2_ = 16; A_3_ = 8; B_1_ = 15.2; B_2_ = 2; B_3_ = 2; L_F_ = 7.2; W_F_ = 1.5; R_0_ = 7.2; G_W_ = 10; T = 1.52 (All units are in millimeters). It needs to be mentioned that other numerical approaches [50] or customized optimization algorithms, such as particle swarm optimization [52], neural networks [53] or other artificial intelligence approaches [54,55] can also be used for further optimization.

### 2.2 Antenna design methodology

**Fig. 3:**
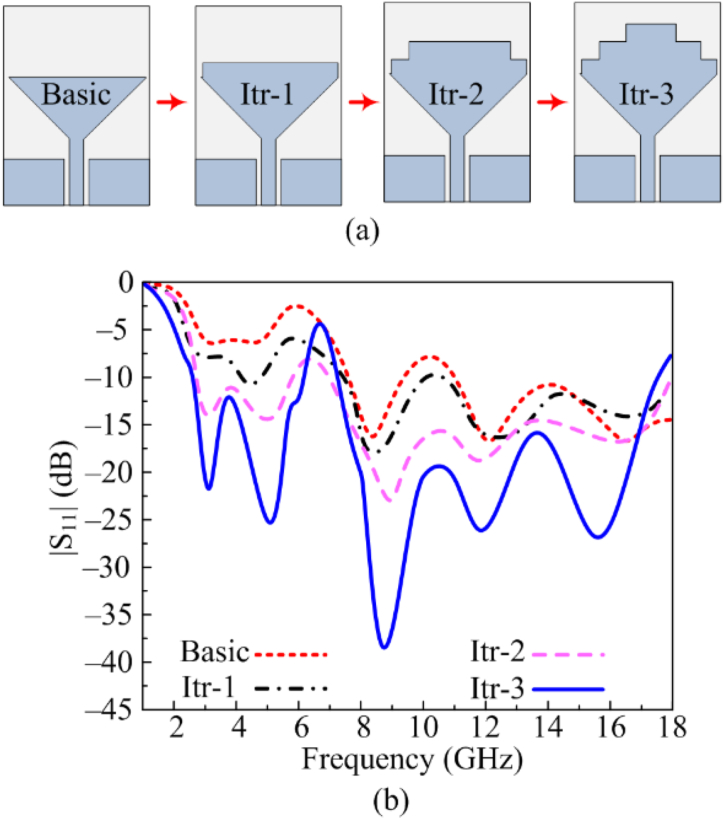
(a) Construction of fractal antenna (b) |S11| results of design steps.

The proposed work with wideband and notch band results is obtained after following four design steps. Each design step and its result in terms of |S11| is given in Fig. 3. In the first stage, a CPW feed antenna with the triangular patch is designed, which has a minor resonance of around 2–4 GHz and 9 GHz. Afterwards, the first iteration is performed by loading a rectangular stub with a length of 16 mm. This results in an increased effective length of the antenna, which consequently reduces the return loss. Afterwards, two more iterations were performed using rectangular stubs of relatively smaller length than the previous steps. With each iteration, as given in Fig. 3(a), the antenna's performance is improved, as illustrated in Fig. 3(b). Thus, the advantage of the staircase structure is utilized, and antenna bandwidth is increased without affecting the overall size of the antenna. As a result, the optimized dual staircase-shaped antenna offers ultrawide bands ranging 2.3–17.8 GHz along with notch band from 6.1 to 7.2 GHz. It is worth noting here that with each consecutive iteration, various parameters of the fractal antenna are optimized to achieve the best possible results.

### 2.3 Parametric analysis

**Fig. 4:**
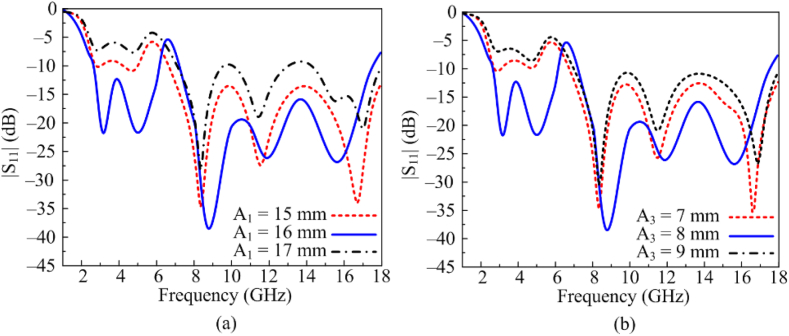
Parametric analysis of various key parameters (a) A_1_ (b) A_3_.

As it is clear from the discussions above, antenna performance is improved by loading stubs. The optimized value of these stubs is obtained after studying and analyzing parametric analysis. The initial stub's length with an ideal value of A1 = 16 mm. Antenna with this value offer dual and wideband frequencies between 2.3 GHz to 6.1 GHz and 7.2–17.8 GHz. The first resonance is impacted when A1 is extended to 17 mm, as seen in Fig. 4. (a). The antenna return loss and bandwidth are once more impacted if the value is decreased from the ideal value and set at 15 mm. The length of the third stub A3 is yet another important element. A dual band response with a wide impedance bandwidth of 3.8 GHz and 10.6 GHz is offered by an 8 mm antenna at its best value, as depicted in Fig. 4 (b). The antenna only gives one wideband with a bandwidth of 8–17.8 GHz when the value is increased and set at 9 mm. Similarly, when value is decreased from optimal value gain the bandwidth and return loss are distorted.

### 2.4 Results and discussion

**Fig. 5:**
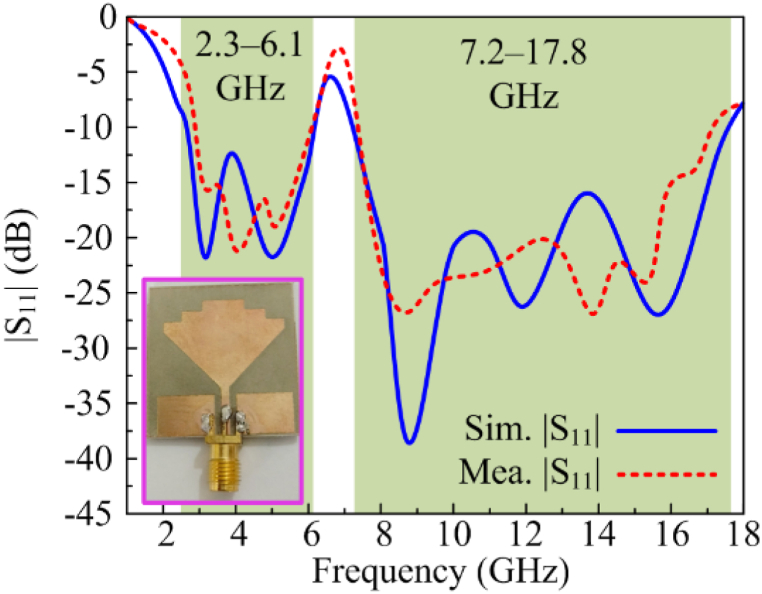
The fabricated prototype was used for testing along with |S11| simulated and measured antenna results.

The proposed result is analyzed in terms of various performance parameters, including |S11|, radiation pattern and gain. For the said purpose, a hardware model of recommended work is manufactured and used for testing and validation of performance parameters, as shown in Fig. 5.

#### 2.4.1 |S_11_|

Within the last decade, various computational methods have been utilized to electromagnetic and microwave designs, such as finite element [56], finite‐difference frequency domain [57], and finite difference time domain [58]. Here, for simulating the S-parameters, Electromagnetic (EM) software tool HFSS v9 is utilized and measured the S-parameter achieved from prototype is given in Fig. 5. It is notable from figure that antenna offer dual-band with |S11| < −10 dB impedance bandwidth ranging from 2.3 to 17.8 GHz coving UWB, extended-UWB, and Ku–band along with globally allocated sub-bands of ISM, WLAN, and 5G sub6–GHz for present and future wireless communicating devices. The antenna also gives notch band along 6.1–7.2 GHz. The figure also shows that the tested and predicated outcomes have strong resemblance, verifying the performance stability in the results. The results' accuracy is due to using a 3D-modeled SMA connector during simulation in EM-software.

#### 2.4.2 Radiation pattern

**Fig. 6:**
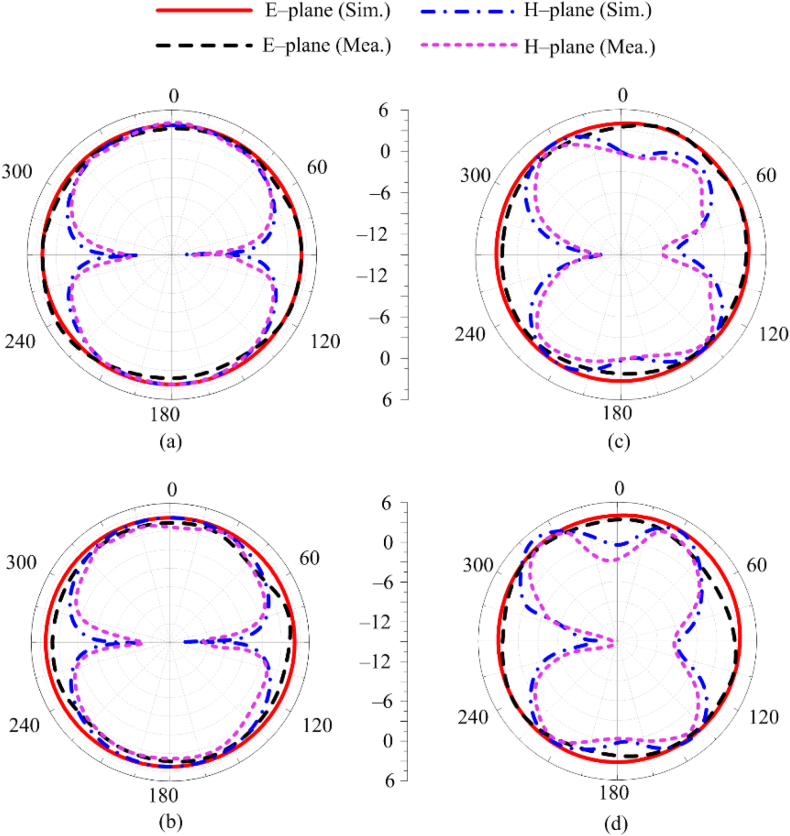
Comparison among radiation patterns at (a) 2.5 GHz (b) 5.4 GHz (c) 8.25 GHz (d) 16 GHz.

The predicated and tested far–field outcomes in terms of the radiation pattern in given in Fig. 6. The radiation pattern of recommended antenna at selective frequencies of 2.5 GHz, 5.4 GHz, 8.25 GHz and 16 GHz is observed in Fig. 6(a), (b), Fig. 6(c), and Fig. 6(d), respectively. The antenna provides an omnidirectional radiation pattern in E–plane for all the resonance frequencies. Yet, the H-plane antenna produces a bidirectional radiation pattern at resonance frequencies. The pattern exhibits some distortion because the electric length is greater than the wavelength at higher frequencies like 8.25 GHz and 16 GHz. Strong agreement between the experimental and simulation outcomes is also shown in Fig. 6. This finding suggests that the antenna is an excellent choice for wireless devices needing an omnidirectional radiation pattern.

#### 2.4.3 Gain and efficiency

**Fig. 7:**
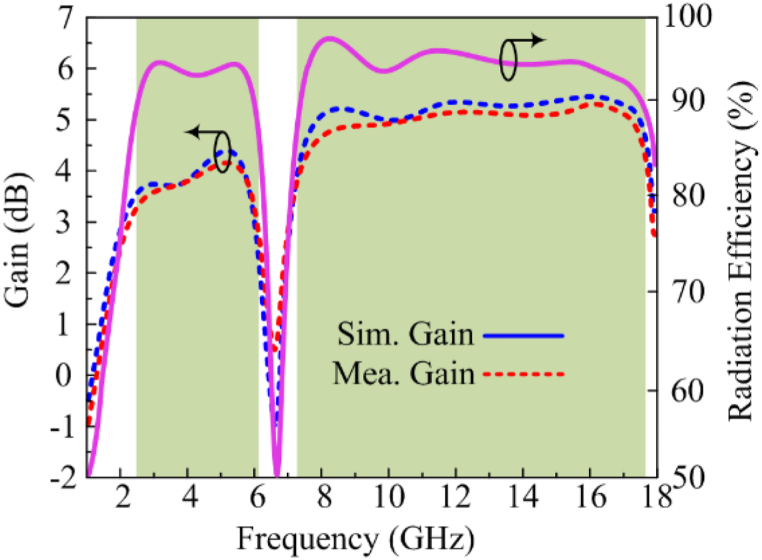
Comparison among gain and estimated radiation efficiency of suggested fractal antenna.

Fig. 7 represents the gain versus frequency of the designed wideband antenna. With a peak value of 4.45 dBi at 5.75 GHz, the antenna gives a gain >3.5 dBi at active bandwidths of 2.3–6.1 GHz. On the opposite side, the antenna provides a gain >4.6 dBi over the second operational bandwidth, which comprises the range of 7.2–17.8 GHz, with a peak value of 5.5 dBi at 12 GHz. The antenna delivers gains of 3.7 dBi, 4.3 dBi, 5.4 dBi, and 5.45 dBi at the intended resonance frequencies of 2.5 GHz, 5.4 GHz, 8.25 GHz, and 16 GHz, respectively. It can be seen that the gain drops to zero at notch band of 6.1–7.2 GHz. The consistency between the tested and estimated results is further expressed in the figure, which suggests that the antenna is a possible applicant for both current and future devices. Moreover, radiation efficiency greater than 90 % is noticed throughout the operation band.

## 3 Proposed MIMO antenna

**Fig. 8:**
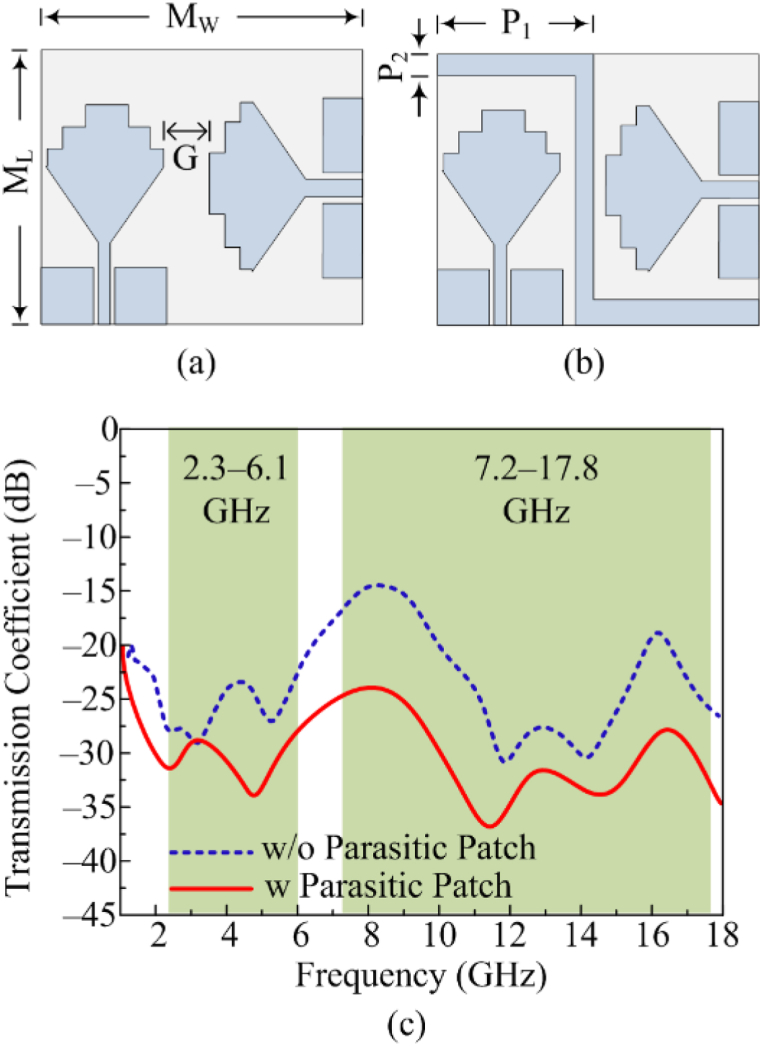
MIMO antenna (a) w/o decoupling structure (b) w-coupling structure; (c) corresponding *trans*-mission coefficient.

Fig. 8(a and b) depicts the MIMO configuration of the proposed dual and wide-band antenna. The two–port MIMO system is adopted to improve the antenna performance for 5G applications. First of all, the geometrical antenna measurement is MW × ML = 57 mm × 34 mm and gap between the two elements is G = 8.5 mm. The antenna offers dual and wide band but minimum isolation of – 15 dB. A parasitic line is inserted in the middle of elements of MIMO antenna to refine isolation to – 25 dB as given in Fig. 8 (c). Each side of parasitic patch has length of P1 = 29 mm and width of P2 = 2 mm. Moreover, a hardware prototype of the proposed antenna is fabricated for measurement to verify the predicated results obtained from software.

### 3.1 MIMO antenna results

This part discusses the output of MIMO antenna loaded with parasitic elements. The results contain the reflection and transmission coefficient, far–filed analysis in terms of radiation pattern and gain, also MIMO parameters in terms of ECC (Envelop Correlation Coefficient), MEG (Mean Effective Gain), CCL (Channel Capacity Loss), and DG (Diversity Gain). At the end of the section, the justification table is provided to compare the recommended work with the state of the art.

#### 3.1.1 S-parameters

**Fig. 9:**
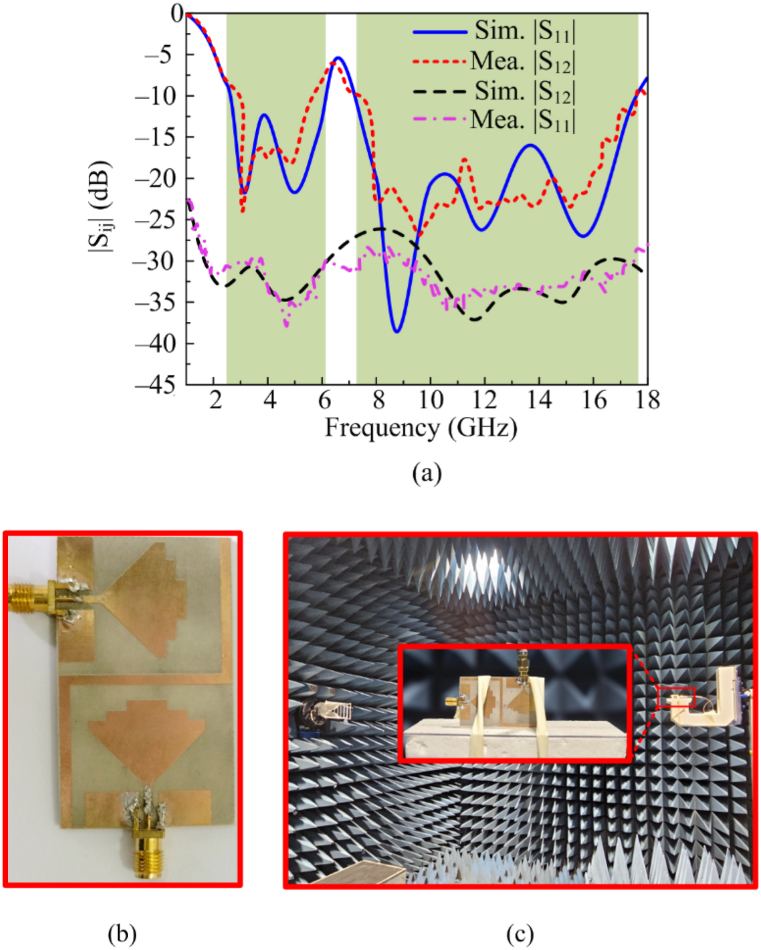
(a) Simulated and tested S-parameters (b) fabricated prototype (c) antenna under test in the chamber.

Fig. 9(a) shows both simulated and tested reflection coefficient plots of the proposed dual and wide-band MIMO antenna. The final geometry of MIMO antenna contains the parasitic patch line to refine the mutual coupling between MIMO elements. The fabricated result of the antenna offers S11 (−10 dB) bandwidth of 2.3–6.1 GHz and 7.2–17.8 GHz with return loss values of < −20 dB and −35 dB, respectively. Similarly, the second element measurement is also made by exiting its corresponding port by connecting 50 Ω loads, offering the same bandwidth and resonance frequency. The antenna covers the overall band of ISM, WLAN, 5G sub–6GHz, C–band, X–band, S–band and Ka–band. The figure also shows the similarity between tested and software-generated results. The operational bandwidth and results make antenna a suitable choice for 5G broadband and compact devices. Fig. 9 (b) depicts the fabricated prototype while the far-field setup is depicted in Fig. 9 (c).

The figure also shows information about the transmission coefficient of the parasitic patch-loaded MIMO antenna. The isolation/mutual coupling is reduced by loading parasitic elements, as discussed in the previous section. The antenna provides very low mutual coupling of a maximum value of −25 dB and a minimum value of −40 dB.

#### 3.1.2 Radiation pattern of MIMO antenna

**Fig. 10:**
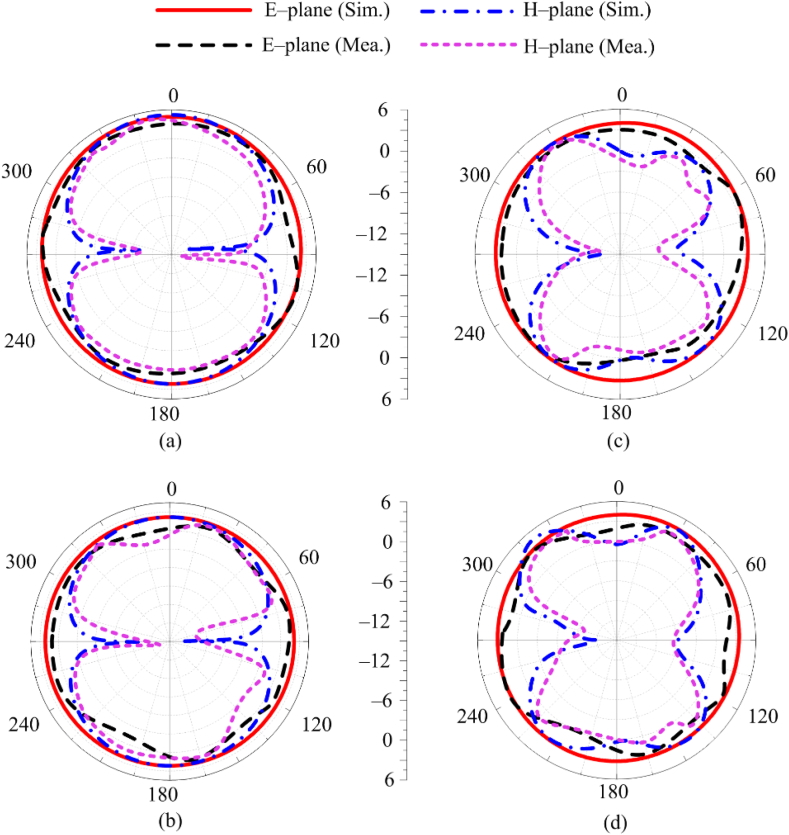
Comparison among radiation pattern of MIMO antenna at (a) 2.5 GHz (b) 5.4 GHz (c) 8.25 GHz (d) 16 GHz.

Fig. 10, The simulated and tested radiation patterns of recommended MIMO antenna for dual and wideband applications. The software generated and tested far–field results in terms of the radiation pattern provided in Fig. 6. The radiation patterns of the antenna at 2.5 GHz, 5.4 GHz, 8.25 GHz, and 16 GHz are plotted in Fig. 10(a–d). As can be seen, the antenna has omnidirectional radiation pattern in the E-plane for all resonant frequencies. In the H-plane, the antenna produces bidirectional radiation patterns. Fig. 10 also demonstrates the consistency of the tested and simulated results.

#### 3.1.3 Envelop Correlation Coefficient

**Fig. 11:**
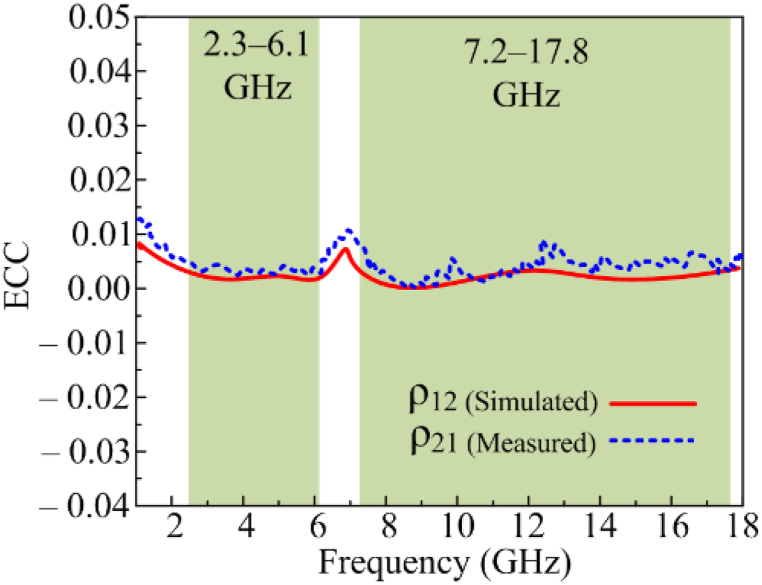
Comparison among estimated and tested ECC.

The Envelop Correlation Coefficient (ECC) of the proposed MIMO antenna is less than −0.01 at operational bandwidth, shown in Fig. 11, which is within the permitted range for MIMO antennas.

#### 3.1.4 Diversity gain

**Fig. 12:**
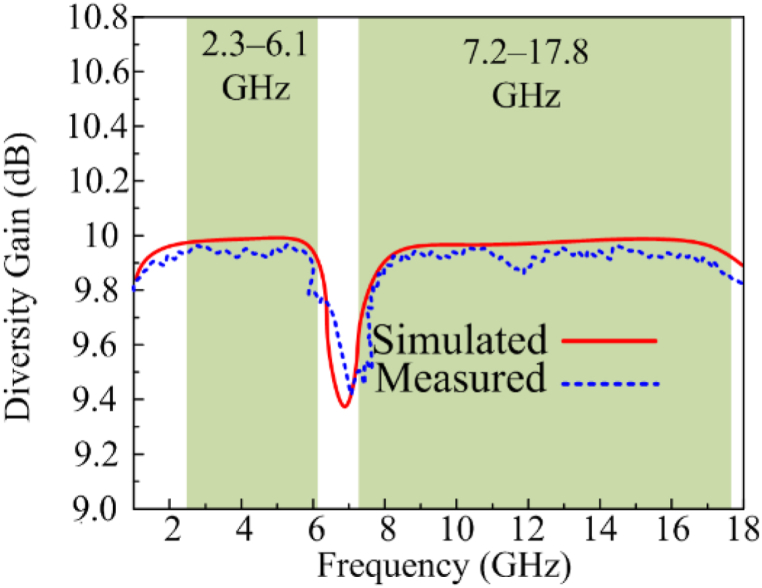
Comparison among estimated and tested diversity gain.

Diversity gain (DG) demonstrates how the diversity scheme of MIMO antennas impacts the radiated power. The diversity gain value is expected to be 10 dB in the ideal situation. According to Fig. 12, the diversity gain for the designed dual and wideband MIMO antenna is approximately 9.99 dB at operational bandwidths of 2.3–3.1 GHz and 7.2–17.8 GHz.

#### 3.1.5 Mean effective gain

**Fig. 13:**
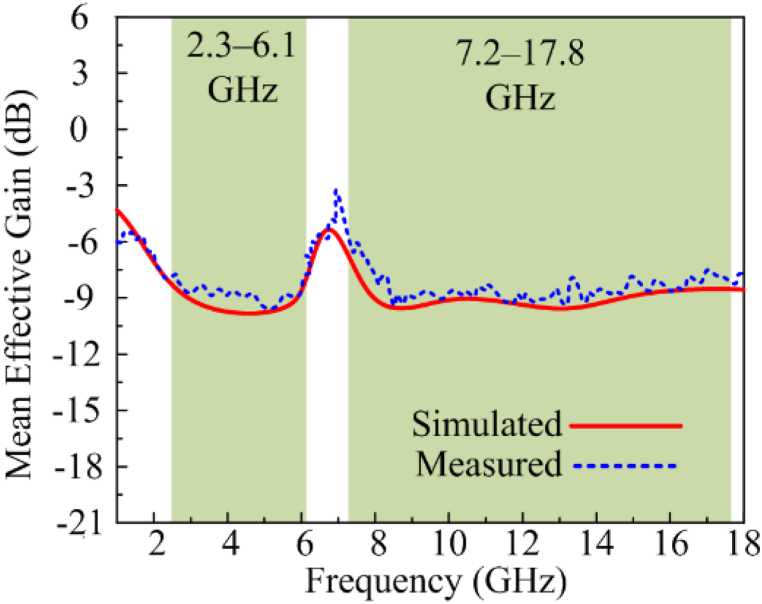
Comparison among estimated and tested MEG.

Mean effective gain is the ratio of the antenna's power received by diversity antenna to its power received by isotropic antenna with a permitted range of −3 to −12 dB. As provided in Fig. 13, the suggested dual and wideband MIMO antenna delivers a mean effective gain of −9 dB for both operational bandwidths, which is within acceptable limits.

#### 3.1.6 Channel capacity loss

**Fig. 14:**
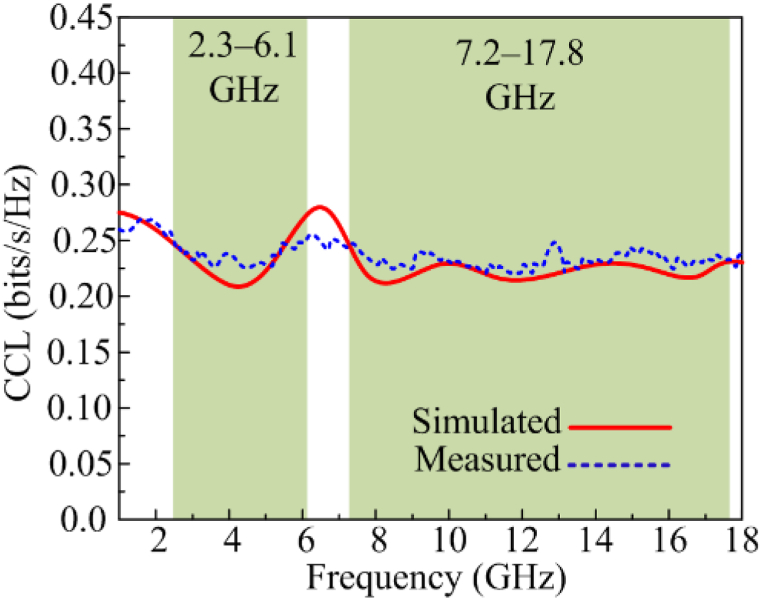
Comparison among estimated and tested CCL.

The estimations of the maximum amount of channel loss that allow the transmission over any communication channel are measured by analyzing channel capacity loss (CCL). The acceptable value of CCL should not be greater than 0.4 bits/s/Hz. Fig. 14, shows that the proposed dual and wideband MIMO antenna offers CCL<0.2 bits/s/Hz, which is under the acceptable range.

#### 3.1.7 SAR analysis

The proposed antenna can also be used for on-body applications; hence SAR (Specific Absorption Rate) analysis is performed. This analysis is crucial, as EM radiation is harmful and may affect human tissues. While analyzing the SAR, the absorption of EM waves is studied according to standards provided by international institutions like IEEE (Institute of Electrical and Electronics Engineers), ICNIRP (International Commission on Non-Ionizing Radiation Protection), and FCC (Federal Communication Commission). These calculations and measurements are performed over the tissue of 1g or 10 g, and the acceptable value of SAR is 2 W/kg for 10g as per Europe, while limit of 1.6 W/kg for 1 g of tissue at US [59].

**Fig. 15:**
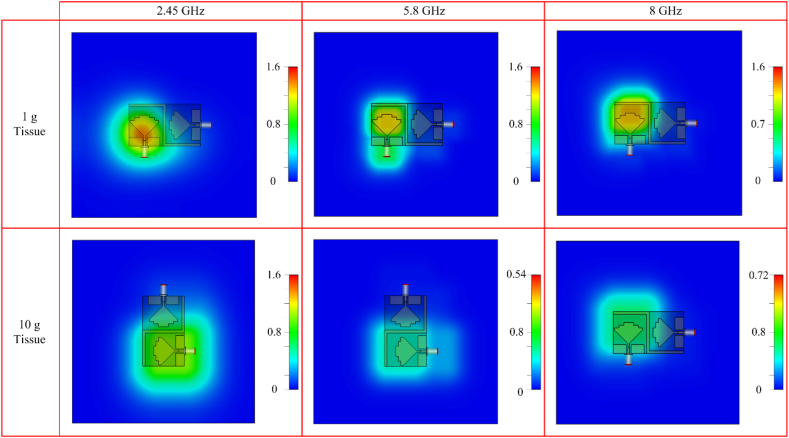
Simulated SAR analysis of the proposed antenna on 4 layered human tissue.

The antenna was placed in our 4-layered human body model with an overall size of 100 mm × 100 mm, with standard skin, fat, muscle and bone thickness of 2 mm, 9 mm, 27.5 mm and 12.5 mm, respectively. The gap between the human model and antenna is fixed to around 5 mm. The study of SAR gives values of 1.43 W/kg, 1.25 W/kg and 1.38 W/kg for 2.45 GHz, 5.8 GHz and 8 GHz for 1 g of tissue. For 10 g of tissue, the antenna provides 0.92 W/kg for 2.45 GHz, 0.54 W/kg for 5.8 GHz and 0.72 W/kg for 8 GHz, as shown in Fig. 15.

## 4 Comparison with state of the art

The proposed antenna offering dual and wideband for future 5G devices is compared with literature work. A number of works are published in literature and explained in literature review. Table 1 has a short conclusion of that explanation. The work operating at targeting frequencies has limitations of either narrow band or large size with complex geometry. The antenna may have limitations of large value of ECC and Dg or low peak gain. The proposed antenna has compact size, with simplified structure with operating over ultra–wide band with low mutual coupling and ECC.

**Table 1:** Suggested work comparison with published works.

Ref	Overall Size (mm × mm × mm)	Bandwidth (GHz)	Mini. Isolation (dB)	ECC	Peak Gain (dBi)	Notch Band	Antenna Type	Technique used
[31]	60 × 60 × 1.52	3–12	>21	0.001	6.64	No	CPW feed patch antenna	Parasitic Decoupler
[32]	38 × 38 × 1.6	2.4–2.7 10–18	>17	0.08	6.2	No	Multi-stub loaded patch antenna	DGS
[33]	55 × 33 × 1.6	3–14	>20	0.02	4.8	Yes	Multiple U slots patch	DGS
[34]	30 × 42 × 079	2.8–11	>20	0.2	5.2	Yes	CSRR loaded Antenna	Strip line + DGS
[35]	39 × 25 × 0.8	2.62–12.5	>20	0.01	5.2	Yes	E-shaped mender structure	Slots + patches loaded
[36]	60 × 60 × 1.6	4–18	>17.5	0.01	6.2	Yes	Modified circular patch	EBG
[37]	34 × 34 × 1.6	2.25–12	>15	0.05	5.5	Yes	Rhombic slot patch	EBG
[41]	43 × 35 × 1.6	3–10.7	>25	0.01	5	Yes	L and U-shaped slot loaded patch	Mushrooms EBG
[42]	60 × 60 × 1.52	3.9–12	>21	0.001	6.94	Yes	Monopole antenna	Parasitic patch
[43]	50 × 82 × 1.6	2.4–13	>20	0.0002	6	Yes	Strip line patch antenna	DGS
Prop. Ant	55 × 33 × 1.52	2.1–18	>45	0.0001	6.5	Yes	Monopole antenna	Parasitic patch

## 5 Conclusion

A compact antenna width wideband, high gain, minimum isolation, and low value of ECC is proposed for future 5G compact devices operating over ISM, WLAN, C and X band applications. The presented antenna offers dual and wide band over 2.3–6.1 GHz and 7.2–17.8 GHz with a peak gain of 4.5 dBi and 6.5 dBi, respectively. The isolation is improved by loading a parasitic patch between antenna elements. The isolation of the antenna is improved from the maximum value of −15 dB to −25 dB. The proposed work offers a low value of ECC, around 0.001 and a good value of DG, around 9.99 dB. The antenna's triangular patch loaded with three stubs gives it a comparatively small size and simplified layout. To increase the antenna's bandwidth and return loss, stubs are introduced. Also, a hardware prototype is created to validate the results of simulations, demonstrating a striking similarity between the two sets of outcomes.

## Data availability statement

Data included in article/supp. Material/referenced in article.

## CRediT authorship contribution statement

**Tanvir Islam:** Writing – original draft, Software, Resources, Formal analysis, Data curation. **Esraa Mousa Ali:** Writing – original draft, Software, Resources, Formal analysis, Data curation. **Wahaj Abbas Awan:** Writing – review & editing, Visualization, Validation, Supervision, Funding acquisition, Conceptualization. **Mohammed S. Alzaidi:** Writing – original draft, Software, Resources, Methodology, Investigation. **Thamer A.H. Alghamdi:** Writing – review & editing, Visualization, Validation, Project administration, Funding acquisition. **Moath Alathbah:** Writing – review & editing, Visualization, Validation, Methodology, Investigation.

## Declaration of competing interest

The authors declare that they have no known competing financial interests or personal relationships that could have appeared to influence the work reported in this paper.
